# Supplementary figures and images for: Peripheral thickening of the sarcomeres and pointed end elongation of the thin filaments are both promoted by SALS and its formin interaction partners
Source: PLoS Genet. 2024 Jan 10;20(1):e1011117. doi: 10.1371/journal.pgen.1011117 (PMC10805286; doi:10.1371/journal.pgen.1011117)

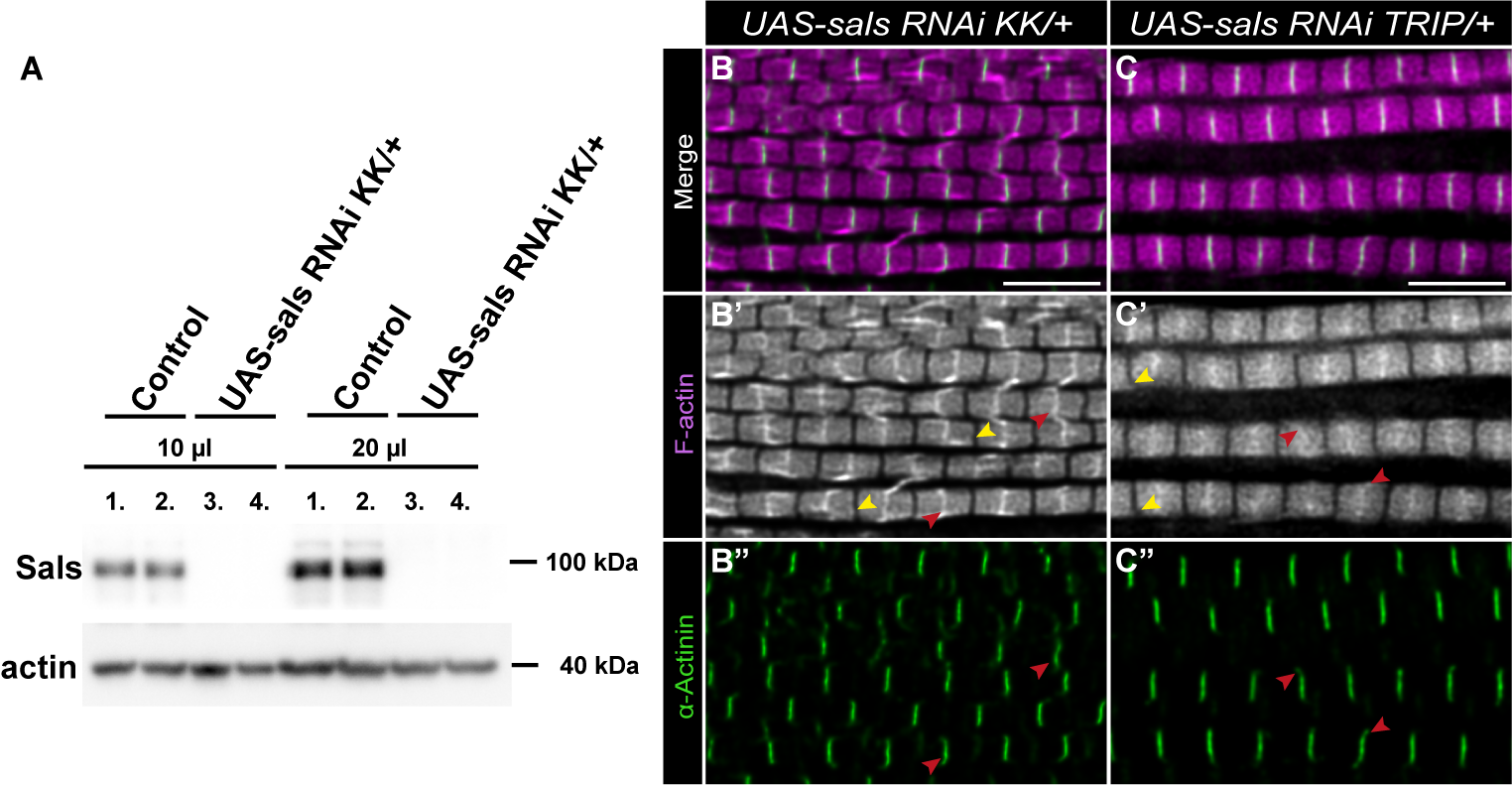

Supplement: S1 Fig — (A) A Western blot analysis is shown, demonstrating that upon sals knockdown with the KK RNAi line level of the SALS protein is strongly reduced. As internal controls, two lanes were loaded for each sample in two volumes (10 and 20 μl), actin was used as loading control. (B-C”) The knockdown of sals either with the KK RNAi line (B-B”) or the TRIP RNAi line (C-C”) resulted in various myofibril defects, including Z-discs deformities (red arrowheads in B’, B”, C’ and C”) and irregular thin filament edges at the H-zone (yellow arrowheads in B’ and C’) as judged by F-actin (magenta in B, C; grey in B’, C’) and α-Actinin (green) staining. Note the stronger effect of the KK line, leading to more severe alterations than the TRIP line. Scale bars: 5 μm. (TIF) [file pgen.1011117.s001.tif]

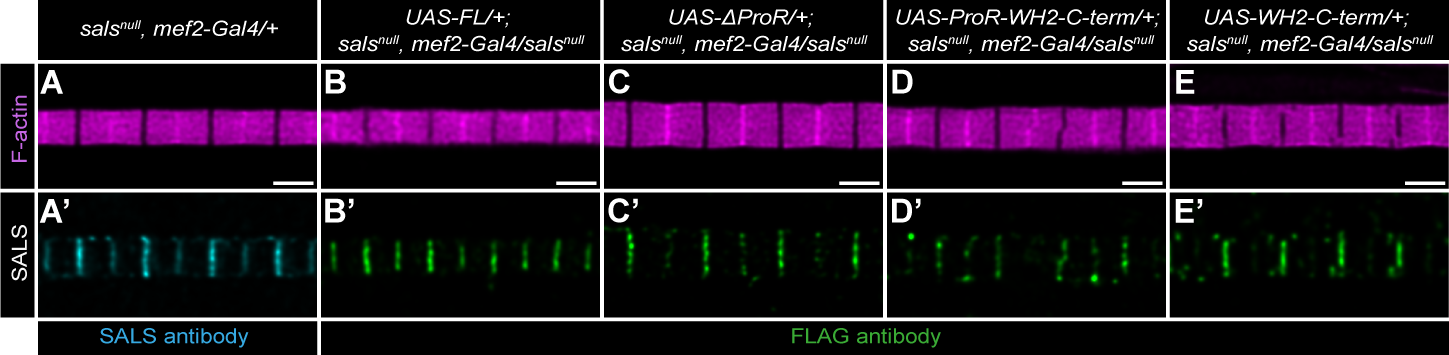

Supplement: S2 Fig — (A-A’) A SALS antibody staining (cyan) in a salsnull, mef2-Gal4/+ (control) adult IFM myofibril reveals a strong accumulation in the H-zone and a weaker signal at the Z-disc. (B-E’) Anti-FLAG staining of myofibrils with the indicated genotype. Note that these myofibrils are derived from rescued flies in which the FLAG-tagged SALS protein forms are expressed in a null mutant background. As expected, the full length (FL) form exhibits a highly similar pattern (B’) as observed with the SALS antibody, and pattern of the ΔProR is also very similar to this (C’). In the cases of ProR-WH2-C-term and WH2-C-term a strong H-zone enrichment is detected, but the signal at the Z-disc signal is either absent or appears weaker (D’, E’) when compared to the FL pattern. Actin (in magenta) was used to highlight the sarcomeres in all samples. Scale bars: 2 μm. (TIF) [file pgen.1011117.s002.tif]

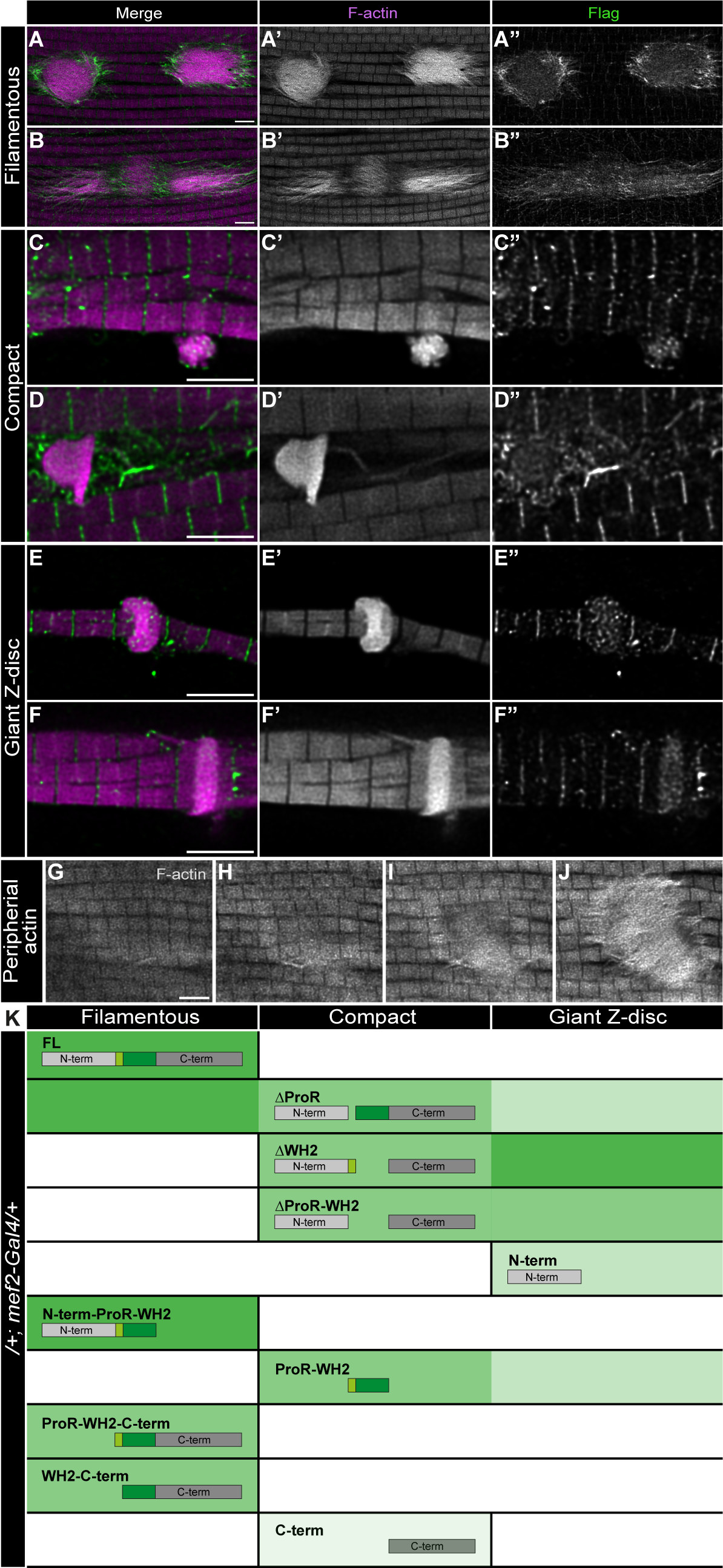

Supplement: S3 Fig — (A-F”) Representation of the diverse actin structures observed upon expression of the SALS isoforms. These actin accumulations can be classified into three major categories: filamentous (A-B”), compact (C-D”) and giant Z-disc-like (E-F”) (two representative examples are shown for each). The SALS isoforms (labelled with a FLAG tag) can be detected within or in the close vicinity of the aggregations (A”, B”, C”, D”, E”, F”) in all cases examined. (G-J) 4 optical Z-sections of the same muscle fiber are shown to illustrate that the actin aggregations typically form in the extra-myofibrillar/peripheral space (images were taken with Z-steps of 0.5 μm). (K) Chart presentation (summary) of the kind of actin structures typically induced by the different SALS isoforms expressed with mef2-Gal4. Opacity level of the green background indicates the penetrance of the phenotype (dark green means high, faint green means negligible occurrence). Scale bars: 5 μm. (TIF) [file pgen.1011117.s003.tif]

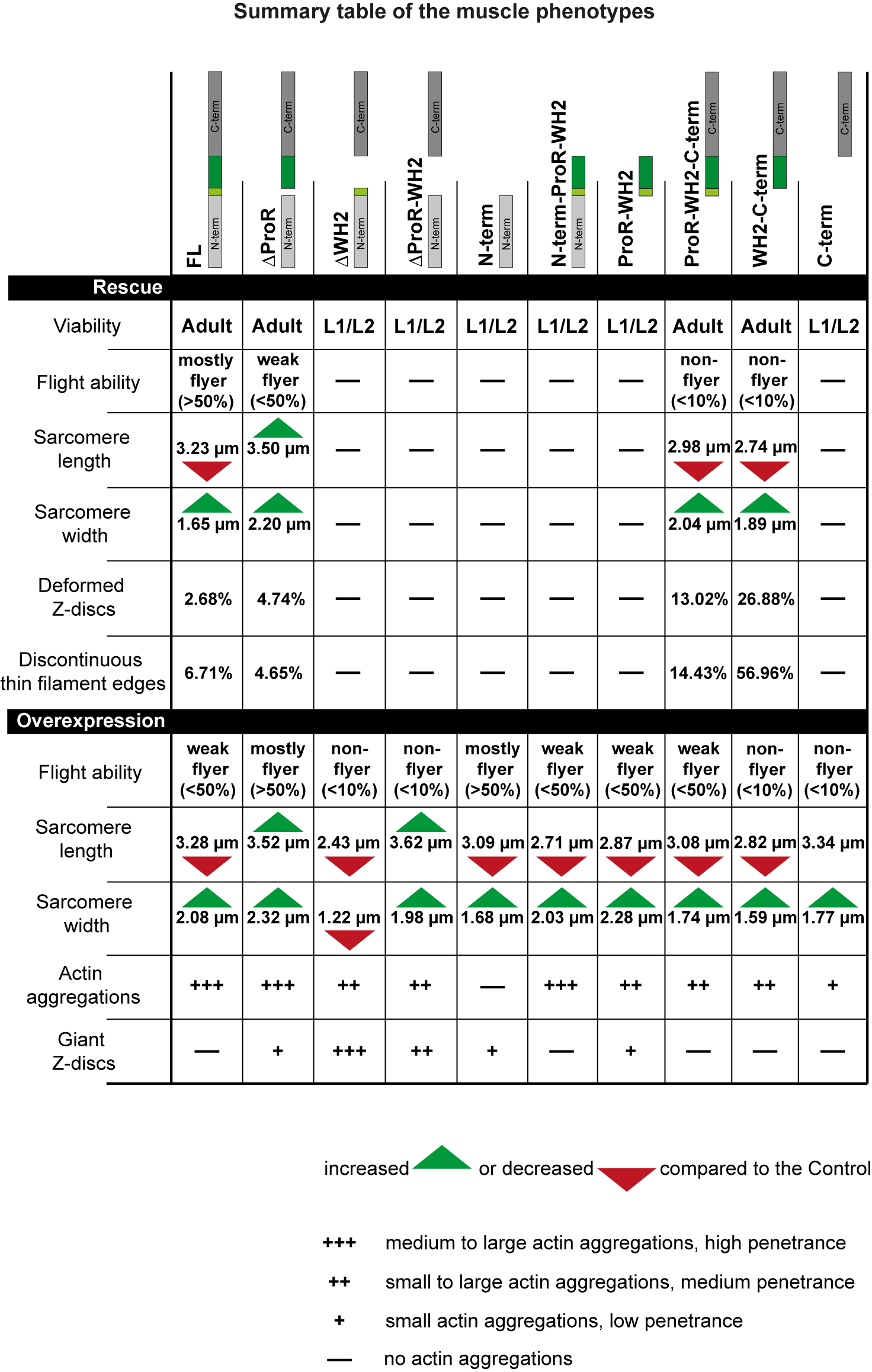

Supplement: S4 Fig — (TIF) [file pgen.1011117.s004.tif]

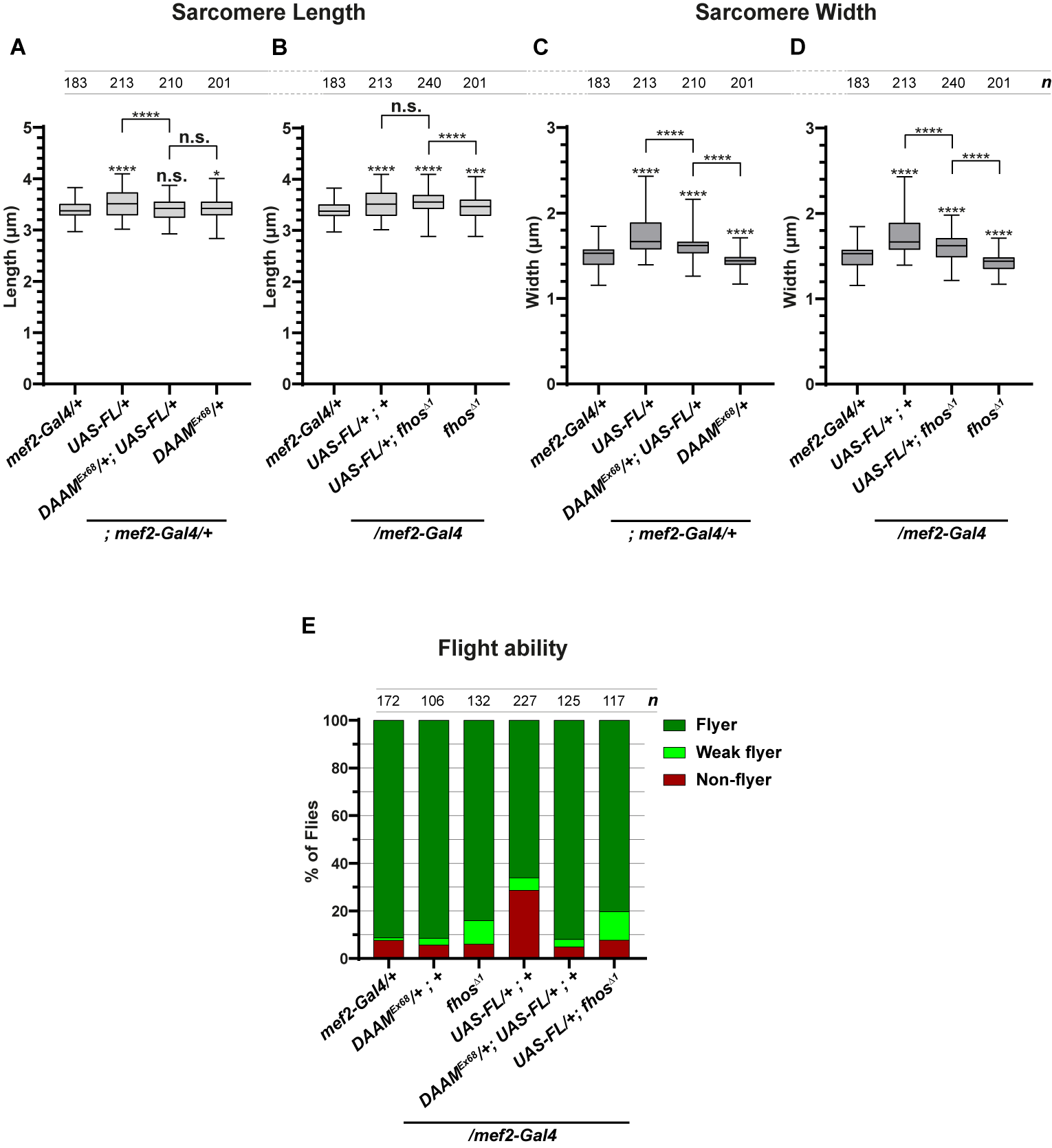

Supplement: S5 Fig — (A-D) Quantification of sarcomere length (A, B) and width (C, D) in control (mef2-Gal4/+) and mutant flies with the indicated genotype (24h AE). P-values were calculated using two-tailed unpaired Student’s t-test with Welch’s correction or Mann-Whitney U test according to the normality (n.s., not significant P>0.05; *P≤0.05; ***P≤0.001; ****P≤0.0001). n indicates the number of sarcomeres measured. (E) Quantification of the flight ability of control and mutant flies with the indicated genotypes (24h AE). Note that the weak flightless phenotype induced by FL-SALS expression is strongly suppressed by presence of the DAAMEx68 and fhosΔ1 null alleles. n indicates the number of flies tested. (TIF) [file pgen.1011117.s005.tif]

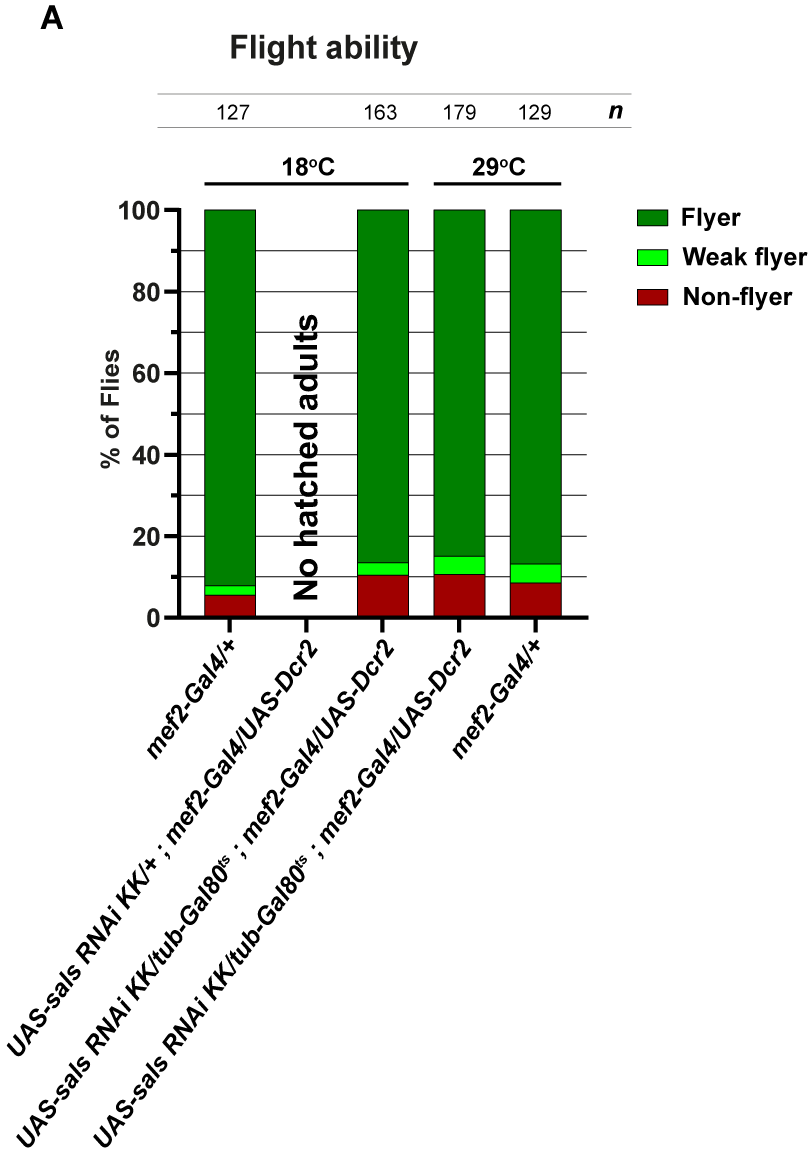

Supplement: S6 Fig — Results of a temperature shift experiment are shown when flies with the indicated genotype were raised at 18 °C until they hatched. Subsequently, half of the adult progeny was kept at 18 °C, whereas the other half was put to 29 °C. After one week of ageing (either at 18 or 29 °C) flight ability was measured. As revealed by this test, flight ability of the tub-Gal80ts/UAS-sals RNAi KK; mef2-Gal4/ UAS-Dcr2 flies was not affected at 29 °C, indicating that SALS is not required to maintain muscle function during adulthood. (TIF) [file pgen.1011117.s006.tif]
