# Supplementary material for: Peripheral thickening of the sarcomeres and pointed end elongation of the thin filaments are both promoted by SALS and its formin interaction partners
Source: PLoS Genet. 2024 Jan 10;20(1):e1011117. doi: 10.1371/journal.pgen.1011117 (PMC10805286; doi:10.1371/journal.pgen.1011117)
Supplement: Attachment — Submitted filename: Rebuttal_Farkas_v1.pdf [file pgen.1011117.s007.pdf]

December 1, 2023

Dear Drs. Cox and Barsh,

Please find attached our revised manuscript (PGENETICS-D-23-00708) entitled " Peripheral thickening of the sarcomeres and pointed end elongation of the thin filaments are both promoted by SALS and its formin interaction partners".

We would like to thank the reviewers for their overall positive assessment of our paper. Reviewer #1 wrote that *"Overall the results as presented are interesting and of a high quality*", Reviewer #2 commented that "*this is a valuable and elegant work that will be of interest to the large muscle and actin regulation audience.*", Reviewer #3 stated that "*It is an important contribution to the muscle field for several reasons: the domain analysis is very carefully done, and especially the interaction with formins sheds new light on the mechanism of Sals during IFM development.*" and that "*Experimental data, images, and statistics are meticulously presented.*", whereas Reviewer #4 summarized our work by writing that "*This well written study moves toward a mechanism that brings together SALS, its various domains, formins, and Tmod in producing appropriately sized sarcomeres.*" and that "*The authors performed an elegant structure-function analysis of SALS*".

Additionally, the reviewers raised several constructive points of criticism as well, all of which are addressed point by point below. We believe that our experimental additions and corrections at numerous places have significantly improved our manuscript, and hope that you share this view.

With kind regards,

József Mihály

**Point by point response to the comments of Reviewer #1:**

A general comment of the Reviewer was that "*My main concern with the manuscript is that it does not significantly advance upon what is already known about the regulation of thin filament and myofibril growth. Nor do the data provide a great deal of novel and mechanistic insight into Sals function. For example, the use of the several different Sals deletion isoforms: while each different isoform has a documented and reproducible phenotype, there is not a*

*clear overall conclusion as to how these phenotypes might arise mechanistically. For the over-expression studies, for example, some isoforms increase or decrease sarcomere length or width, but there is no clear conclusion made by the authors,"*

We think that there is no clear definition of what is or what would be a "*significant advance upon the regulation of thin filament and myofibril growth*". In our view, the work described in this paper represents a considerable (if not a significant) advance because we provide new insights into the mechanisms of myofibril growth, as well as into SALS function. Regarding the general mechanisms of thin filament and myofibril growth, there are at least two critical aspects that remained poorly understood, i.e. the mechanisms of peripheral/radial growth of the myofibrils and that of the pointed-end elongation of the thin filaments. As to radial growth, our work led to two important advances, (1) we identified SALS as a new player with a role in this process, (2) several lines of evidence was collected suggesting that radial growth of the sarcomeres is initiated at the Z-disc. As to pointed-end elongation, our new data provides further support for an annealing mechanism. Regarding SALS function, we report that, beyond the previously described role in sarcomere elongation, SALS is involved in Z-disc formation and peripheral growth of the sarcomeres. In addition, we revealed that the roles of SALS in sarcomere elongation and radial growth are both dependent on formin type of actin assembly factors, and we think that collectively, these data clearly represent novel mechanistic insights. Moreover, we report one additional novelty, notably that the excess of SALS promotes the formation of actin aggregates highly resembling to those described in nemaline myopathy patients. Therefore, although we recognize that uncovering the details of the molecular mechanisms is not in the focus of our paper, we are still convinced that this body of work includes numerous novel findings that will advance our knowledge and make a significant contribution to the field.

1. There is also a concern that the authors do not track the accumulation of the mutant isoforms in some key experiments. I note that Figure S1 shows (rather faint) accumulation of the FLAG-tagged isoforms in a wild-type background, but it would be valuable to understand the stability and assembly properties of the mutant isoforms in a Sals null or knockdown background. This would seem to provide critical insight into mechanisms of Sals function.

We agree with the Reviewer that protein localization is an important piece of information as to the interpretation of phenotypic rescue experiments, in particular when rescue is carried out with truncated protein isoforms. Although we had these data in hands already at the initial submission of our manuscript, these were left out to avoid extreme lengthiness. Now we include these data (see Figure S2) for the 4 constructs providing rescue of the null mutant till adulthood. In this condition the FLAG tagged FL-SALS construct displays a strong accumulation in the H-zone and a weaker staining at the Z-disc in the IFM myofibrils, which highly resembles the expression pattern of the wild type endogenous protein. As compared to this, the *ΔProR* construct exhibits a similar pattern with a somewhat weaker overall intensity. The *ProR-WH2-C-term* and *WH2-C-term* forms also enrich in the H-zone, however, their accumulation at the Z-disc is much weaker than in the case of FL-SALS. These data indicate that the truncated proteins providing the best rescue (i.e. till adulthood) kept the ability to integrate into the myofibrils in a somewhat lower but still in a comparable amount as the wild type protein. In addition, we revealed a notable difference between the *ΔProR,* and the *ProR-WH2-C-term* and *WH2-C-term* forms, because the accumulation pattern of *ΔProR* is closer to wild type and it provides a significantly better rescue of the flight ability

than the other two truncated forms (Figure 3C). Therefore, we propose that in the absence of the N-term region (*ProR-WH2-C-term* and *WH2-C-term* constructs) Z-disc accumulation of the protein is partly impaired, resulting in the formation of largely non-functional myofibrils often displaying irregularities in the thin filament order both at the Z-disc and in the H-zone.

A summary of these localization data is now included in the revised ms, and a supplementary figure is added to depict the staining data.

2. In Figure 4D, the authors indicate that over-expression of full-length Sals significantly affects sarcomere length, however this is not consistent with the text (line 224-225).

Corrected accordingly.

**Point by point response to the comments of Reviewer #2:**

Major :

1. RNAi Sals phenotypes (Fig. 1 and 2) are based on the analyses of a single RNAi line and without checking the level of Sals expression in the IFM in the RNAi context. A second sals RNAi line needs to be tested to avoid potential non-specific RNAi effects. Assessing the level of Sals expression in the IFMs will help to evaluate the strength of RNAi knockdown.

Use of the VDRC KK RNAi line was already reported by the laboratories of Eyal Schejter, Ben-Zion Shilo and Richard Cripps (Shwartz et al., 2016; Czajkowski et al., 2021). Moreover, we note that the RNAi phenotypes we (and others) found were highly similar to the LOF phenotypes reported by Bai et al., 2007. On this ground, non-specific RNAi effects appeared very unlikely. In line with this, Western blot analysis of the SALS protein level upon UH3-Gal4 driven knockdown of *sals* with the KK RNAi line revealed a very strong reduction in SALS levels (Figure S1). In addition, to fulfill the request of the Reviewer, we tested another RNAi line of a different origin (BDSC TRIP collection, #JF01109), resulting in a somewhat weaker but qualitatively similar effects as the KK line (see revised Figure 1).

2. Observation that excess of Sals leads to generation of actin aggregates and giant Z discs is interesting, however stating that Sals overexpression could represent novel nemaline myopathy model is not appropriate considering that Sals has no orthologous gene in Human.

Whereas it is undoubtedly true that *sals* has no orthologous gene in human, it also remains true that according to the current view nemaline myopathy is primarily caused by a sarcomeric actin imbalance, that can be induced by either LOF or GOF conditions of at least a dozen of genes (including Lmod3, considered as a functional ortholog of SALS). Taking these together with our observation that upon SALS overexpression the phenotypic traits exhibit remarkable parallels to the effects described in muscles of patients with nemaline myopathy, we strongly believe that a SALS overexpression system could represent a novel NM model

because it appears that the key in such a model is actin imbalance, and not gene specificity. Yet, we've tried to somewhat tone down our statement as to this concern.

Minor :

1. The term « isoforms » for the generated SALS constructs producing truncated proteins is not well adapted

The term "isoform" was replaced for another phrase at all places.

2. Fig. 4D shows that overexpression of FL SALS has significant negative effect on sarcomere length – authors comment in the line 224 is contradictory

Indeed, the effect appeared statistically significant, yet still very mild. The sentence has been corrected to ensure consistence.

**Point by point response to the comments of Reviewer #3:**

1. The sals RNAi knockdown in Fig. 1 should be better described in the results section (partial knockdown of Sals protein with reference, specificity with reference to previous papers)

Use of the VDRC KK RNAi line was already reported by the laboratories of Eyal Schejter, Ben-Zion Shilo and Richard Cripps (Shwartz et al., 2016; Czajkowski et al., 2021). However, specificity of the line was not reported in those studies. Nevertheless, the RNAi phenotypes (we and others found) are similar to the phenotypes of the LOF allele (Bai et al., 2007), clearly arguing for a specific effect. To further clarify this issue, we tested the KK RNAi line with Western blot analysis, and we revealed a very strong reduction in the SALS protein level in the IFM (Figure S1). As an additional specificity test, we examined another RNAi line of a different origin (BDSC TRIP collection, #JF01109), resulting in somewhat weaker but qualitatively similar effects as the KK line (see revised Figure 1).

Grammar and orthography should be carefully checked; some examples are given below:

38: subject to

61: we found that excess amounts

258: what is "cancellated"?

412: previous studies, not "former studies"

We thank the Reviewer for the corrections; "cancellated actin accumulations" was corrected for "loosely organized, actin-based meshworks".

**Point by point response to the comments of Reviewer #4:**

(1) How would Tmod overexpression affect the increased sarcomere length and width seen when (delta)ProR or (delta)ProR-WH2 is overexpressed? This experiment may provide insight to whether this domain interacts with Tmod in some way to restrict length of the sarcomere.

We addressed this question by using ΔProR, and we found that Tmod overexpression has no effect on sarcomere length when expressed together with ΔProR (3.320±0.212 µm n=171 for *UAS-ΔProR* versus 3.319±0.251 µm n=264 for *UAS-Tmod*; *UAS-ΔProR*; mean±SD), whereas the concomitant OE results in thicker sarcomeres than the ones observed upon ΔProR expression (1.797±0.292 µm n=264 versus 1.575±0.234 µm n=171, mean±SD). These results might suggest that the ProR domain is involved in Tmod interaction to restrict sarcomere length. Nevertheless, we do not consider these findings convincing enough to include them in the manuscript, because the presence of ΔProR suppresses the sarcomere shortening effect of Tmod OE. In addition, because the *UAS-Tmod*; *mef2-Gal4* combination is lethal, this set of studies could only be carried with *UH3-Gal4*, which is viable when combined with the *UAS-Tmod*. Note, however, the difference between the Gal4 drivers: OE of ΔProR with *mef2-Gal4* resulted in a significant increase in sarcomere length (Figure 6M), whereas the OE with *UH3-Gal4* had no effect on length, indicating that this phenotype is very sensitive to protein expression levels. Therefore, we think that alternative approaches will be required to investigate this potential interaction, and for the time being, we suggest to leave them out from the manuscript.

(2) It is curious that the aggregates are only seen when SALS is overexpressed when NM cases are typically caused by loss of function mutations. Many of the reported mutations are in regions other than the WH2 domain according to Yuen et al., 2014. As Leiomodin is considered to be an actin nucleator (Chereau et al., 2008); how do the authors reconcile this with the results of this study that suggest that SALS is not itself adding to the thin filament?

Although in the case of Lmod3 the NM-linked mutations are indeed considered as LOF type mutations, and many of the other mutations affecting the NM genes are also classified as LOF alleles, a significant number of the NM mutations exhibit an autosomal dominant inheritance (reviewed e.g. in Laitila and Wallgren-Pettersson, 2021), suggesting that GOF effects can also contribute to the muscle weakness in the NM cases. It follows that the current view on NM is that the primary cause is an imbalance in actin regulation, which is often the consequence of a LOF type mutation, but sometimes it is due to GOF effects. Consistent with this view, in the cases of ACTA1 and NEB, the two main NM genes in human, the LOF and GOF situations both cause NM-like alterations in muscle structure in a

zebrafish model (Sztal et al., 2015). Therefore, our finding that the actin aggregates are only seen when SALS is in excess, is largely in line with current literature, and it is also in harmony with the actin imbalance model of NM.

As to the molecular mechanisms of Lmod and SALS, we need to emphasize that despite initially being recognized as an actin nucleation factor, in these days Lmod is viewed as a multifunctional protein, acting as a pointed-end capper and promoting thin filament elongation with an unknown mechanism in muscles (nicely reviewed e.g. in Tolkatchev et al., 2021, FEBS J.). Given that the SALS protein also accumulates at the pointed-end, and it promotes filament elongation (with an unknown mechanism), the parallels to the Lmod family remain striking. In fact, it is the *in vitro* documented actin nucleator function of Lmod that is difficult to reconcile with its *in vivo* function during muscle development.

(a) NM is typically diagnosed following confirmation of actin rods/aggregates on histology and electron microscopy. What do the aggregates look like via TEM in the overexpression condition compared to the deformed/shortened Z discs seen in TEM of SALS RNAi in Figure 2. When do these aggregates first occur? Do more aggreggates get added over time?

We thank the Reviewer for the suggestion to provide a developmental analysis of the actin aggregations. These studies were carried out with two constructs (ΔWH2 and ΔProR-WH2) frequently causing the formation of actin aggregations. The aggregations were counted in the period between 30h APF and 24h AE at four time points. Confocal analysis revealed that the aggregations first appear between 30-48h APF, and they remain detectable till adulthood. To provide a quantitative assessment, the number of the giant Z-discs was compared at a pupal and an adult timepoint, leading to the conclusion that there is no significant increase in the number of this type of aggregation over time. Quantification of the less dense, net-like aggregations was not straightforward, yet we didn’t notice an obvious change in their occurrence.

As suggested by the Reviewer, the actin-based aggregations were also analyzed with TEM. Consistent with the variability observed with confocal microscopy, we detected several types of electron-dense aggregations on the TEM sections, that varied in size, shape and orientation. In addition, the presence of large zebra bodies or zebra body-like structures was also evident. Collectively, the plethora of these different types of aggregations (see several examples below) highly resemble the histopathological diversity of the actin aggregations associated with NM and NM related disorders. A brief description of these new findings is now included in the revised ms, and Figure 5 has been modified accordingly.

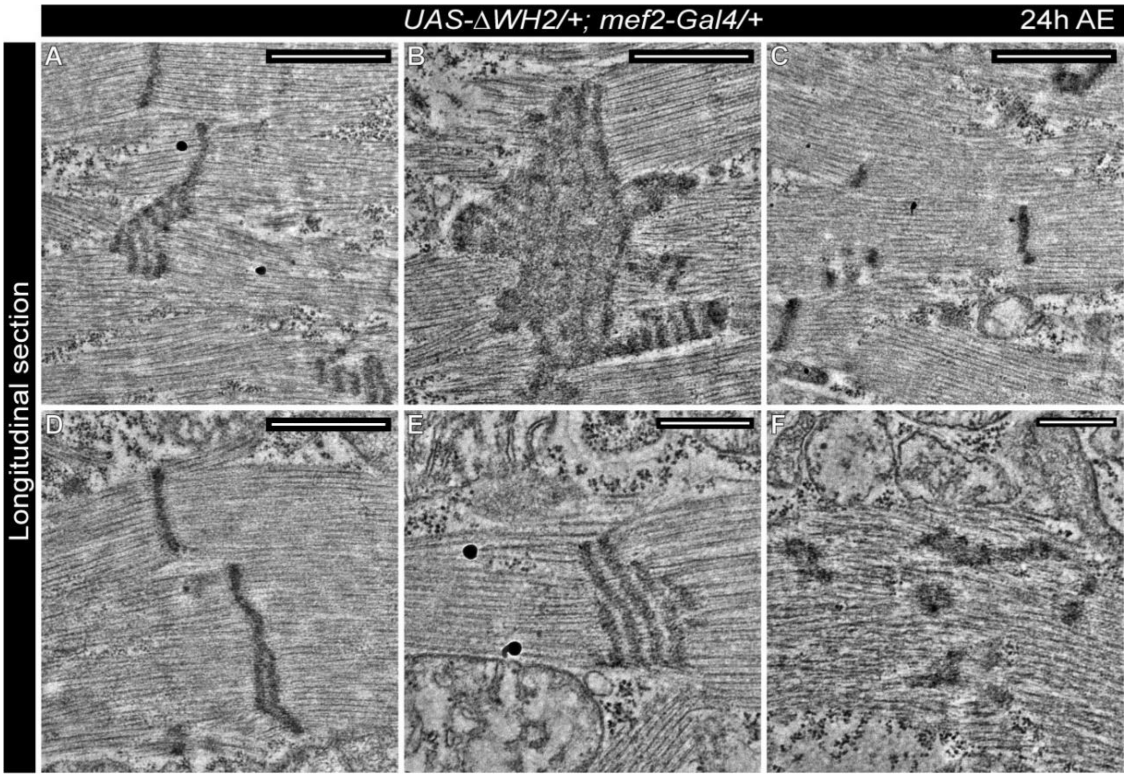


*TEM images illustrating the various types of electron dense aggregations upon (delta)WH2 overexpression. Note the zebra bodies on A,B,E and F; the huge aggregation on B, and the numerous smaller ones on C and F. Scale bars: 1 μm (A-D), 500 nm (E,F).*

(b) The authors describe some of the aggregates as occurring "mostly in the extra-myofibrillar space in the peripheral region of the myofibers" and that, with the exception of (delta)WH2 overexpression, they did not disrupt gross myofibrillar organization. In the SALS RNAi, the organization was disrupted in the peripheral region. Example images of these peripheral aggregates in the overexpression condition would be helpful. Could the authors suggest what makes the peripheral region more "vulnerable" when radial growth seems to be affected throughout.

To better illustrate the peripheral aggregates, we now included additional panels in Figure S3 (formerly S1), which is meant to help to appreciate that these aggregations are located in the extra-myofibrillar space. As to the differential effect of silencing versus overexpression on the peripheral region of the myofibrils, we suggest that the myofibrillar versus extra-myofibrillar effects should be considered independent of each other: the silencing of *sals* reduces myofibril width but it has no obvious effect on organization of the extra-myofibrillar space, as compared to this, the overexpression of (some forms of) SALS increases myofibril width, and additionally, it promotes the formation of actin aggregates in the extra-myofibrillar space. One possible mechanism could be that upon SALS overexpression, part of the excess SALS is able to integrate into the myofibrils (and induces radial growth), whereas the other part remains in the extra-myofibrillar space where it succeeds to promote actin assembly, presumably by interacting with additional actin regulatory factors.

(c) When the formins were knocked down in the SALS overexpression lines, were the aggregates still observed?

Based on examination of the FL and ΔProR constructs, the aggregations are still present upon DAAM or Fhos knock down (see Figure below). Thus, it appears that unlike myofibril growth, formation of the (mostly) extra-myofibrillar actin aggregations is not formin dependent. Alternatively, it is also possible that the formins act redundantly in the cytoplasmic space, which is not the case in the myofibrils.

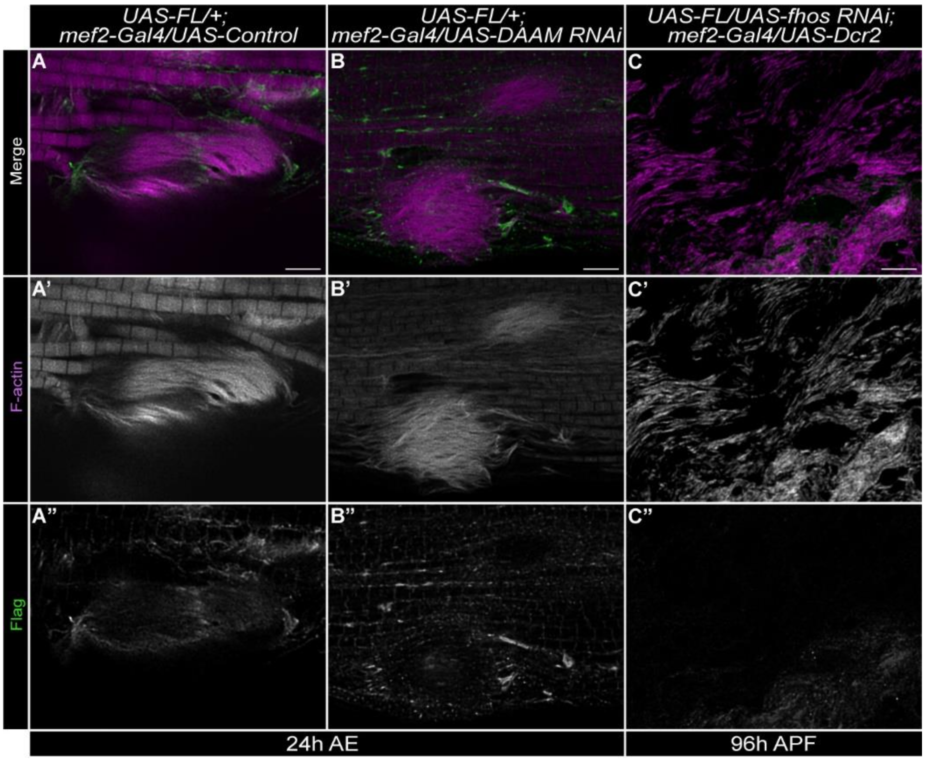


(3) Several clarifications are needed about the nanoscopy experiments.

The requested clarifications are provided below.

(a) Figure 8 would benefit from a diagram representing how this localization pattern maps onto the sarcomere. It is challenging to visualize because the orientation is different compared to the rest of the figure.

We rotated the images on panel C with 90 degrees to warrant uniform orientation of the image panels.

(b) In prior work done by some of the authors using the same nanoscopy technique (Szikora et al, JCB 2019), both Fhos and DAAM are shown to be closer to the M line than SALS or Tmod at 24h AE. How does this fit in with the model presented in this manuscript, where thin filament elongation is dependent on these formins, yet the formins "lead the way" in front of even the N-terminus of SALS.

The dSTORM localization data published by us indicated a roughly 20 nm distance between SALS and the formins. A possible interpretation of this observation is that formins might promote the formation of short (20-40 nm long) actin oligomers in a narrow stripe along the M-line, and these short F-actin polymers will subsequently anneal to the pointed-end with the possible assistance of SALS. Thus, this model predicts that the main function of SALS is to facilitate the annealing step, whereas assembly of the actin oligomers would be an entirely formin dependent process. Consequently, in the absence of the formins SALS would not be able to promote pointed-end elongation, and one would expect that the effect of SALS on sarcomere (thin filament) length would be completely formin dependent, much the same way as our data indicated it (Figure 6).

In addition, although the high points of the SALS and formin signals do not colocalize, a partial overlap is still present.

(c) In the previous publication, the authors show the localization of SALS at the Z line at 24h AE. In the text of this manuscript, the authors state that "the accumulation of SALS in the Z-discs of growing sarcomeres was too weak to enable STORM analysis with sufficient precision." Does this statement refer to the pupal stage examined at 72h APF in Figure 8? Could the same technique not be applied at a later pupal stage (96h APF) or at 0h AE to get a better idea of what is happening at the Z disc with SALS? Having any information about if the localization or level of SALS changes would bolster the authors' claim that SALS is critical for Z disc formation.

Immunofluorescent microscopy is often limited by antibody quality, and it is also true for fluorescent nanoscopy. The SALS antibody (Bai et al., 2007), targeting the central region of the protein, exhibits high quality and generates a strong signal. It detects SALS in both the H-zone and Z-disc of both developing and mature myofibrils (see figure below), hence the presence of SALS at the Z-disc during the pupal stages, is not in question. On the other hand, we found that the FLAG signal, used to detect the N and C-terminal regions of the protein, is significantly weaker (for no obvious reason). Despite this, as most of the SALS signal (~70-80%) originates from the H-zone (see figure below), it remains sufficient for consistent measurements in both growing and mature sarcomeres in the H-zone, but not at the Z-disc where the signal is weak. In summary, we encountered challenges in producing reliable measurements at the Z-disc when using the FLAG antibody in this specific case, nevertheless, the results with the SALS antibody allow us to safely conclude that the protein is present at the Z-disc in the adult and pupal muscles as well.

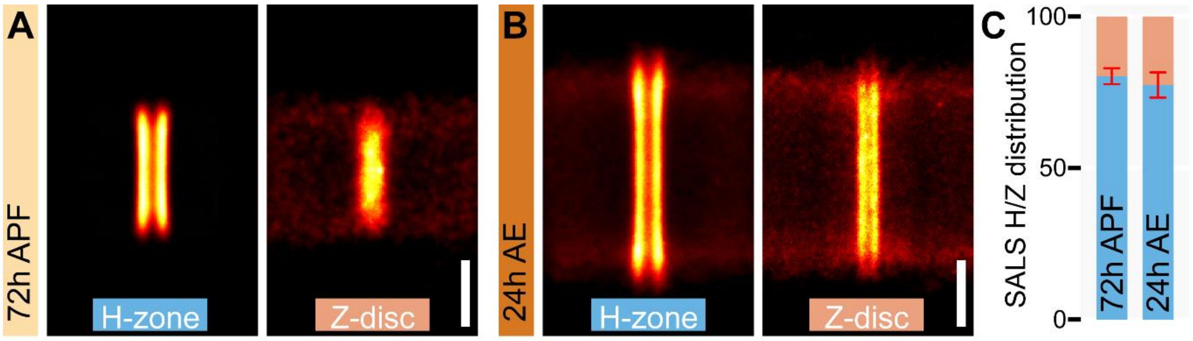


*A SALS antibody staining revealed that the protein is primarily localized into the H-zone during the interval covered by our nanoscopic assessment, and it is also present in the Z-disc (quantified in C). In mature sarcomeres, SALS exhibits a "double line" distribution in the Z-disc (B), with an average Z-line peak distance of 46.7 nm, positioning it in close proximity of the barbed ends of the thin filaments. In growing sarcomeres (72h APF), SALS shows a more diffuse accumulation around the Z-disk (A). While not included in this figure, confocal microscopic examinations revealed that a few days after hatching, most of the SALS signal is found in the I-band (with a very faint staining in the H-zone).*

(d) For Figure 9C, what is localization density? The methods section does not clearly define this term, it simply states that "absolute number of localizations" was measured.

In essence, it is an intensity-based measurement employed to estimate relative protein levels. However, to clarify the concept of "localization density", we revised the relevant paragraph in the methods section.

(4) The authors suggest that SALS is not required for maintenance of the adult sarcomeres. To prove this point, the authors should remove SALS after growth of the sarcomeres is completed, using the GAL80ts system.

We followed the suggestion of the Reviewer, and employed the Gal80ts system to investigate the role of *sals* during muscle/myofibril maintenance. As a control, we raised *mef2-Gal4*/+ flies at 18 °C until they hatched as adults, subsequently half of the adult progeny was kept at 18 °C, whereas the other half was put to 29 °C. Ratio of the flightless flies was measured after one week in both cases, resulting in a 5.5% penetrance at 18 °C and 8.5% at 29 °C. When a similar experiment was carried out with *tubGal80ts; UAS-salsRNAiKK; mef2-Gal4* flies, ratio of the flightless individuals was 10.4% at 18 °C and 10.6% at 29 °C, indicating that SALS is not required for maintenance of the adult muscle functions, and hence, myofibril structure. As a control for activity of the tubGal80ts, we raised *UAS-salsRNAiKK; mef2-Gal4* flies either in the presence or absence of *tubGal80ts* at 18 °C, revealing a 100% larval lethality in the absence of *tubGal80ts*, and 100% adult viability in the presence of *tubGal80ts* (Figure S6). These data are now included in the ms, to back up the suggestion that SALS is not required for maintenance of the adult sarcomeres.

(5) The authors find that SALS is required for Z disc assembly with is required for radial growth. They speculate that SALS does this by "promoting the assembly of Z-disc integrated actin filaments". To support this, the authors could examine genetic interactions between a-actinin and SALS at the Z disc.

We followed this suggestion, and a dominant genetic interaction test was carried out with a null allele of α-actinin (*Actn*$^{8}$). As shown below, heterozygosity for *Actn*$^{8}$ mildly increased the ratio of the impaired Z-discs present upon *sals* knock down (*UH3-Gal4/+; UAS-salsRNAi KK*), whereas it had no significant effect on myofibril width. Thus, this experiment alone did not provide a strong support for the idea of "*SALS promoting the assembly of Z-disc integrated actin filaments*", nor did it rule it out. We conclude that further experiments will be required to answer this question by investigating α-actinin with alternative approaches, and by also investigating other potential actin regulators and crosslinkers. These far more thorough studies are, however, beyond the scope of the current analysis.

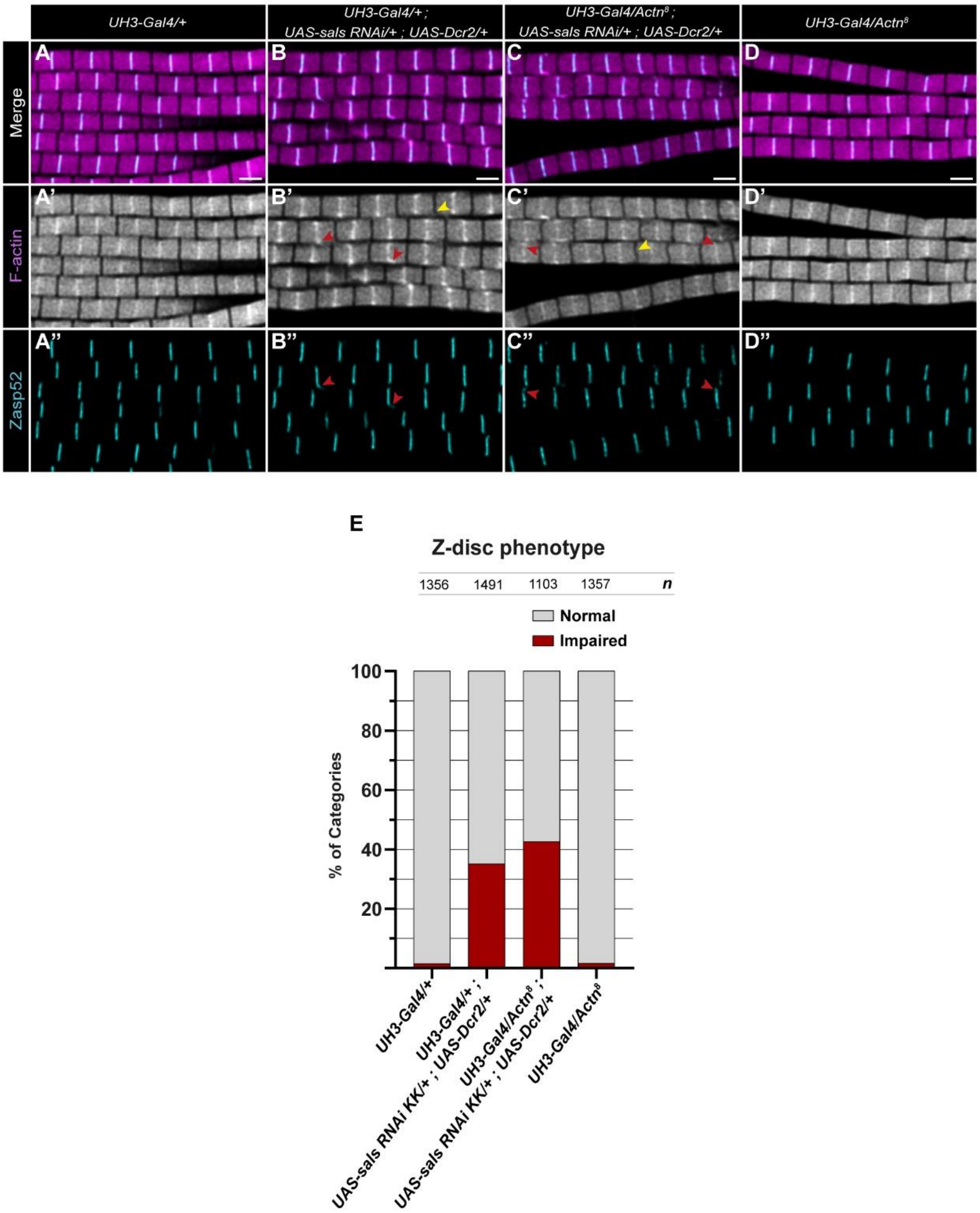


(6) Additional Technical concerns/comments

a) In Figure 2, "mutant" is used rather than knockdown when referring to the RNAi condition.

Corrected accordingly.

b) The authors use GAL4/+ as the control. The standard in the field is to use, for example, GAL4/UAS-mCherry expression of a gene and GAL4/ UAS-mCherry RNAi for RNAi transgenes. This is important particularly for experiments in which the authors are driving two trangenes; as an example, the proper control for Figure 6 in which a UAS-forminRNAi and a SALS construct are expressed would be UAS-GFP RNAi and that SALS construct and a UAS-GFP and the UAS-formin RNAi. The authors may find that with multiple UAS's, the phenotypes may be altered. This reviewer recognizes that this would require redoing multiple figures. Perhaps just showing that the UAS-SALS constructs still provide the same phenotypes in terms of expressivity and penetrance as a supplemental figure would suffice.

We share the view of the Reviewer that using equal number of UAS elements is more appropriate to obtain a reliable dataset, and for this reason such controls were already available at the initial submission of our paper. However, because presence of the UAS-control lines did not find significantly alter sarcomere size in the adult IFM, we initially decided to use the *mef2-Gal4*/+ control at most places. Given that in our practice the expression of fluorescent proteins sometimes affected cell viability, we avoided using such controls. Instead, we worked with *UAS-Dicer2* and *UAS-Vang*, two UAS transgenes which exhibited either no or very mild effects on adult sarcomere length and width when expressed with muscle drivers (and of which *UAS-Vang* was integrated into the VIE-260B landing site, also used for generation of the SALS transgenes). These controls are now included in Figure 6 and Figure 7, summarizing three sets of experiments with multiple UAS constructs. Note that in the case of the 96h pupal samples, the *UAS-Vang/+; mef2-Gal4/UAS-Dicer2* control displayed somewhat longer and thicker sarcomeres than we have seen in the formerly used *mef2-Gal4/UAS-Dicer2* control. Nevertheless, the robust effect of *fhos* knock down is still evident, and we can conclude that presence of the multiple UAS's did not have a considerable modifying effect on the main sarcomeric parameters we followed.

c) Labeling— the authors use FL or FL/+ UAS-DAMM… The FL is a UAS construct; it is unclear and misleading to have all the UAS constructs called out differently.

It is a fair comment that there was some inconsistence in our labeling. The main reason behind was that at some points we tried to simplify the otherwise complicated and long genetic nomenclature. We have now revised the labeling in multiple figures (and at multiple places) to indicate the genotypes in a more precise and uniform way.

d) The authors should include a Summary Table of SALS constructs (like in Fig 3) but with additional headings such as viability, S length, width, radial, Z-disc, aggregates, with ++ to denote the effects for each construct.

A Summary Table is now provided (as Figure S4), as suggested by this Reviewer.
